# Patient-reported health-related quality of life, work productivity, and activity impairment during treatment with ALO-02 (extended-release oxycodone and sequestered naltrexone) for moderate-to-severe chronic low back pain

**DOI:** 10.1186/s12955-017-0749-y

**Published:** 2017-10-17

**Authors:** Arnold J. Weil, Elizabeth T. Masters, Alexandra I. Barsdorf, Almasa Bass, Glenn Pixton, Jacquelyn G. Wilson, Gernot Wolfram

**Affiliations:** 1 Non-Surgical Orthopaedics, PC, 335 Roselane St NW, Marietta, GA 30067 USA; 2 Pfizer Inc, 235 E 42nd St, New York, NY 10017 USA; 3 Pfizer Inc, 4222 Emperor Boulevard, Suite 335, Durham, NC 27703 USA

**Keywords:** Abuse-deterrent opioids, Patient-reported outcomes, Chronic low back pain, Naltrexone, Oxycodone and extended-release opioid

## Abstract

**Background:**

The efficacy of ALO-02, an abuse-deterrent formulation containing extended-release oxycodone and sequestered naltrexone, in the treatment of chronic low back pain (CLBP) was studied in a 12-week randomized controlled trial. Primary efficacy endpoint results have been published previously (Rauck et al., 2015). The current paper focuses on patient-reported outcomes for health-related quality of life (HRQL), work productivity, and activity impairment that were assessed during this study.

**Methods:**

This was a double-blind, placebo-controlled, randomized withdrawal study in patients with moderate-to-severe CLBP. After a screening period (≤2 weeks), patients entered an open-label titration period (4–6 weeks). Treatment responders were then randomized to a double-blind placebo-controlled treatment period (12 weeks). HRQL was assessed using changes in the Short Form-36 v2 Health Survey (SF-36v2) and the EuroQol-5 Dimensions Health Questionnaire 3-Level version (EQ-5D-3L). Work productivity and regular activities were evaluated using the Work Productivity and Activity Impairment Questionnaire: Specific Health Problem (WPAI:SHP).

**Results:**

A total of 410 patients received ALO-02 during the open-label titration period, of which 280 (intent-to-treat (ITT) population) were treated during the double-blind placebo-controlled treatment period (placebo, n = 134; ALO-02, n = 146). Significant improvement was observed for all SF-36v2 subscales and component scores (*p* < 0.005) and the EQ-5D-3L summary index and visual analog scale (*p* < 0.0001) during the titration period. Improvement was also significant (*p* < 0.0001) for all WPAI:SHP outcomes except ‘work time missed due to CLBP’ for the titration period. Significant differences favoring ALO-02 compared with placebo were only observed for the SF-36v2 Bodily Pain subscale (*p* ≤ 0.0232; ITT population) during the double-blind treatment period and the overall study period (screening to the end of the double-blind treatment period). The percentage change in activity impairment due to low back pain subscale of the WPAI:SHP significantly favored ALO-02 compared with placebo for the ITT population when considering the overall study period (*p* = 0.0040).

**Conclusions:**

HRQL, work productivity, and activity impairment may be improved with ALO-02 treatment.

**Trial registration:**

ClinicalTrials.gov NCT01571362, registered April 3, 2012.

## Background

Chronic pain is a major public health challenge owing, in part, to its prevalence and economic burden [1]. In 2012, approximately 25 million Americans were living with chronic pain, which resulted in reduced health-related quality of life (HRQL) and work productivity [2, 3]. The economic impact of chronic pain has been estimated to be $560–635 billion per year in 2010 US dollars [1].

The long-term management of chronic pain with opioids should only be initiated when other pain treatments, such as non-opioid pain medicines or immediate-release opioid medicines, are ineffective or are not tolerated [4, 5]. Guidelines developed by the American Pain Society, the American Academy of Pain Medicine, and the Centers for Disease Control and Prevention stress the importance of patient assessment and monitoring to avoid opioid misuse or abuse [5, 6]. Despite these measures, opioid abuse is a recognized problem that is approaching epidemic proportions [7].

Abuse-deterrent opioids (ADOs) have been developed as part of a multifaceted approach to enable safer use of opioids [8]. ADOs include formulations that impede tampering by physical (e.g., crush-resistant) or chemical (e.g., opioid receptor antagonist) means [8]. Designed as an ADO, ALO-02 capsules comprise pellets of extended-release oxycodone hydrochloride and sequestered naltrexone hydrochloride, an opioid receptor antagonist.

The efficacy and safety results of a phase III clinical trial to evaluate the analgesic efficacy of ALO-02, when compared with placebo, in patients with moderate-to-severe chronic low back pain (CLBP) have been published [9]. This study showed that treatment with ALO-02 was superior to placebo for low back pain as measured on the 11-point Numeric Rating Scale (NRS)-Pain. Similar results were also seen on the Brief Pain Inventory-Short Form (BPI-sf). Patients treated with ALO-02 reported improvement during the open-label titration period on other assessments such as the Patient’s Global Assessment (PGA) of Low Back Pain and Roland Morris Disability Questionnaire (RMDQ). Satisfaction with Treatment favored ALO-02 over placebo. Overall, this study found that ALO-02 was efficacious in treating CLBP and presented a safety profile similar to that of other opioids [9].

The current paper focuses on secondary objectives of this study that relate to additional patient-reported outcomes of ALO-02 in treating CLBP. Assessments of HRQL, work productivity, and activity impairment in patients treated with ALO-02 for the management of moderate-to-severe CLBP are reported here.

## Methods

This was a double-blind, placebo-controlled, randomized withdrawal study in patients from the United States with moderate-to-severe CLBP (ClinicalTrials.gov: NCT01571362). Informed consent and institutional review board approval were obtained before study initiation.

### Study population

Patients (aged ≥18 years) with a documented diagnosis of nonspecific moderate-to-severe CLBP present for at least three months requiring continuous around-the-clock opioid analgesic treatment and an average daily NRS-Pain score for low back pain of ≥5 and ≤9 were included in the study. Major exclusion criteria were history (within the past 2 years) of lumbosacral radiculopathy, spinal stenosis, documented drug or alcohol abuse, or a positive urine drug test for illicit substances or opioid medications not prescribed to the patient.

### Study design

This study consisted of four periods, starting with an initial screening period lasting ≤2 weeks during which standard medical assessments were conducted to determine patient eligibility. Eligible patients entered an open-label ALO-02 conversion titration period (4–6 weeks) during which pain management was individualized by titrating ALO-02. At the end of the open-label titration period, patients tolerating ALO-02 and with NRS-Pain scores of 4 or lower were then randomized to either continue on active ALO-02 treatment or placebo. Patients were randomized in a 1:1 ratio using an interactive voice or web response system to receive a randomization number and blinded treatment assignment. Randomization was stratified by prior pain analgesic (opioid or non-opioid) and study center. Patients, investigators and clinic personnel were blinded to the randomized drug. The double-blind randomization period included a two-week blinded taper for both the treatment and placebo arms to ensure integrity of blinded data, followed by 10 weeks of treatment with ALO-02 or placebo. During the two-week post-treatment follow-up period, patients were tapered off ALO-02 and converted to standard of care treatment.

### Patient-reported outcome assessments

HRQL was measured using the Short Form-36v2 Health Survey (SF-36v2) [10] and the EuroQol-5 Dimensions Health Questionnaire 3-Level version (EQ-5D-3L) [11, 12].

The SF-36v2 is a self-administered questionnaire measuring physical functioning, role limitations due to physical problems, social functioning, bodily pain, mental health, role limitations due to emotional problems, vitality, and general health perception. These eight domains also combine to form two component summary scores, the Physical Component Summary and the Mental Component Summary. Scores range from 0 to 100, with higher scores indicating better health states.

The EQ-5D-3L is a self-completed standardized instrument used as a generic measure of health status using a simple descriptive profile consisting of five dimensions (mobility, self-care, usual activities, pain/discomfort, anxiety/depression), each of which is assessed on a three-level severity scale (no problems, some or moderate problems, extreme problems). These scores are combined to form a single index utility value with higher scores indicating better health. Additionally, a standard vertical 20 cm visual analog scale (VAS) was used to record an individual’s rating of their current health status.

Work productivity and activity impairment were measured using the Work Productivity and Activity Impairment Questionnaire: Specific Health Problem (WPAI:SHP) [13]. The WPAI:SHP questionnaire measured the effect of a specific health problem (i.e. low back pain) on work productivity and activity impairment. Specific outcomes were absenteeism (work time missed), presenteeism (impairment while working), overall work impairment (absenteeism plus presenteeism), and activity impairment (impairment in regular activities) due to CLBP. Each score is represented as a percentage, with higher scores indicating less productivity or greater impairment.

### Endpoints

The endpoints assessed were mean changes in the SF-36v2, EQ-5D-3L, and the WPAI:SHP from screening period to the end of titration period (open-label titration period), from randomization baseline to the end of study (double-blind treatment period), and from screening period to the end of double-blind treatment period (overall study period).

### Statistical analyses

Analysis of patient-reported outcomes was conducted on all patients during the titration period and on the intent-to-treat (ITT) population, consisting of all randomized patients who received at least one dose of study medication after randomization. For changes during the open-label titration period, a paired *t* test was used on observed data. For changes during the double-blind treatment period and overall study period, analysis of covariance was used, with treatment and prior pain analgesic (opioid or non-opioid) as categorical factors and the screening or baseline score and final total daily dose of the titration period (for double-blind treatment period analysis only) as covariates. Last observation carried forward was used for missing data at the end of the treatment or study period.

## Results

### Patients

Of the 410 patients who received ALO-02 during the open-label titration period, 280 (ITT population) were treated during the double-blind treatment period (placebo, *n* = 134; ALO-02, *n* = 146). Detailed demographic data from this study have been published [9]. During the open-label titration period, 56.8% of patients were female, and patients had a mean (SD) age of 50.1 (12.48) years and a mean (SD) body mass index of 30.2 (5.46) kg/m^2^. Of the patients randomized to the double-blind treatment period, 57.5% were non-opioid users prior to the start of this study. These patients reported a mean NRS-Pain score of 7.1 and a mean BPI-sf score of 6.7 at screening. Overall, patient demographics were similar between the placebo and ALO-02 groups. The most common reasons for patients not entering the double-blind treatment period were AEs (13.9%) and “did not meet entrance criteria” (10.0%), which included those unable to tolerate ALO-02 and not meeting treatment response criteria for randomization.

### Patient-reported outcomes

During the open-label titration period, changes on the SF-36v2 showed statistically significant improvement for all subscales for all patients (*p* ≤ 0.0026) and ITT patients (*p* < 0.0001; Table 1). Statistically significant improvement was also observed for the EQ-5D-3L summary index and VAS for all patients and ITT patients (*p* < 0.0001 for both populations). Improvement was also statistically significant for all patients (*p* < 0.0001) and the ITT population (*p* ≤ 0.0017) for all WPAI:SHP outcomes, except the work time missed due to low back pain subscale for all patients (*p* = 0.3822).

**Table 1:** Change in patient-reported outcomes – titration period

Instrument	All patients		ITT patients	
	Mean change	*p*-value	Mean change	*p*-value
Short Form-36v2 Health Survey^a^				
Physical Functioning	6.7	<0.0001	8.0	<0.0001
Role-Physical	7.7	<0.0001	9.1	<0.0001
Bodily Pain	9.8	<0.0001	11.6	<0.0001
General Health	2.3	<0.0001	3.1	<0.0001
Vitality	4.6	<0.0001	5.8	<0.0001
Social Functioning	5.0	<0.0001	7.0	<0.0001
Role-Emotional	3.4	<0.0001	4.7	<0.0001
Mental Health	2.3	<0.0001	3.5	<0.0001
Physical Component Score	8.2	<0.0001	9.6	<0.0001
Mental Component Score	1.6	0.0026	2.9	<0.0001
EuroQol-5 Dimensions 3-Level^b^				
Summary Index	0.12	<0.0001	0.14	<0.0001
Visual Analog Scale	8.16	<0.0001	9.84	<0.0001
Work Productivity and Activity Impairment Questionnaire: Specific Health Problem^c^				
Percentage work time missed due to low back pain	−1.3	0.3822	−4.9	0.0017
Percentage impairment while working due to low back pain	−22.4	<0.0001	−25.9	<0.0001
Percentage overall work impairment due to low back pain	−21.6	<0.0001	−26.9	<0.0001
Percentage activity impairment due to low back pain	−27.2	<0.0001	−32.0	<0.0001

*ITT* intent to treat

^a^Higher scores indicate a better health-related quality of life. ^b^Higher scores indicate a better health. ^c^Higher scores indicate less productivity/greater impairment

During the double-blind treatment period, the difference between patients randomized to placebo and patients randomized to continue ALO-02 for the mean change in SF-36v2 was statistically significant only for the bodily pain subscale (*p* = 0.0100; Table 2). No statistically significant treatment differences were observed for changes in the EQ-5D-3L summary index and VAS, or the WPAI:SHP.

**Table 2:** Change in patient-reported outcomes – double-blind treatment period

Instrument	LS Mean Change			
	Placebo	ALO-02	Difference^a^	*p*-value
Short Form-36v2 Health Survey				
Physical Functioning	−2.06	−1.44	0.62	0.5181
Role-Physical	−2.56	−2.59	−0.03	0.9733
Bodily Pain	−5.07	−2.69	2.37	0.0100
General Health	−1.55	−2.10	−0.55	0.4712
Vitality	−1.62	−2.77	−1.16	0.2898
Social Functioning	−3.17	−1.69	1.48	0.1565
Role-Emotional	−2.57	−3.44	−0.86	0.5220
Mental Health	−2.95	−2.93	0.02	0.9865
Physical Component Score	−2.70	−1.68	1.02	0.2491
Mental Component Score	−2.29	−2.98	−0.69	0.5219
EuroQol 5-Dimensions 3-Level				
Summary Index	−0.061	−0.029	0.032	0.0605
Visual Analog Scale	−3.61	−2.89	0.72	0.7196
Work Productivity and Activity Impairment Questionnaire: Specific Health Problem				
Percentage work time missed due to low back pain	6.49	3.72	−2.77	0.4944
Percentage impairment while working due to low back pain	6.77	4.39	−2.38	0.6008
Percentage overall work impairment due to low back pain	10.85	5.71	−5.14	0.3604
Percentage activity impairment due to low back pain	10.56	6.41	−4.15	0.1031

*ALO-02* extended-release oxycodone and sequestered naltrexone, *LS* least squares

^a^Difference calculated as ALO-02 minus placebo; a positive difference favors ALO-02, and a negative difference favors placebo for SF-36v2 and EQ-5D-3L; a negative difference favors ALO-02 for WPAI:SHP

For the overall study period, the difference between patients randomized to placebo and patients randomized to continue ALO-02 for the mean change in SF-36v2 was statistically significant only for the bodily pain subscale (*p* = 0.0232; Table 3). There were no statistically significant differences between placebo and ALO-02 in either the EQ-5D-3L summary index or VAS. The WPAI:SHP showed a statistically significant difference only for mean change in percentage of activity impairment due to low back pain (*p* = 0.0040) for patients treated with ALO-02 compared with those on placebo.

**Table 3:** Change in patient-reported outcomes – overall study period

Instrument	LS Mean Change			
	Placebo	ALO-02	Difference^a^	*p*-value
Short Form-36v2 Health Survey				
Physical Functioning	5.32	6.84	1.52	0.1731
Role-Physical	5.68	6.78	1.10	0.3290
Bodily Pain	6.39	8.78	2.39	0.0232
General Health	1.28	1.07	−0.20	0.8139
Vitality	3.46	3.01	−0.45	0.6878
Social Functioning	3.58	5.57	2.00	0.0658
Role-Emotional	0.99	1.22	0.23	0.8670
Mental Health	0.09	0.47	0.37	0.7259
Physical Component Score	6.42	8.09	1.67	0.0989
Mental Component Score	−0.44	−0.45	0.00	0.9969
EuroQol 5-Dimensions-3 Level				
Summary Index	0.085	0.106	0.021	0.2280
Visual Analog Scale	4.75	7.01	2.26	0.2701
Work Productivity and Activity Impairment Questionnaire: Specific Health Problem				
Percentage work time missed due to low back pain	−1.00	−2.09	−1.09	0.7480
Percentage impairment while working due to low back pain	−16.18	−25.38	−9.20	0.0581
Percentage overall work impairment due to low back pain	−15.16	−25.51	−10.35	0.0679
Percentage activity impairment due to low back pain	−18.62	−26.81	−8.19	0.0040

*ALO-02* extended-release oxycodone and sequestered naltrexone, *LS* least squares

^a^Difference calculated as ALO-02 minus placebo; a positive difference favors ALO-02 and a negative difference favors placebo for SF-36v2 and EQ-5D-3L; a negative difference favors ALO-02 for WPAI:SHP

## Discussion

Patient-reported outcomes are useful in evaluating treatment efficacy and interpreting clinical outcomes [14]. This study assessed these outcomes in a chronic pain population enriched by treatment responders. A randomized withdrawal trial design is recommended by the FDA to reduce placebo exposure and minimize high dropout rates associated with clinical trials of opioids. This design may be especially suitable to test reformulations of approved opioids [15]. The Initiative on Methods, Measurement, and Pain Assessment in Clinical Trials (IMMPACT) guidelines suggest this trial design may increase assay sensitivity [16]. The results of the open-label titration period may provide valuable insights since it is thought to closely mimic clinical practice [17].

The primary efficacy and safety data from this study showed that ALO-02 was superior to placebo in reducing pain in patients with CLBP and exhibited a safety profile similar to that of other opioids [9]. In the current analysis, patients reported significant improvement in HRQL, work productivity, and activity impairment with ALO-02 during the open-label titration period, which parallels the significant pain reduction associated with CLBP observed after ALO-02 administration during this period [9]. The change in the EQ-5D summary index from screening to the end of the open-label titration period of 0.14 is above a previously reported mean minimal important difference of 0.074 (range, −0.011–0.140), indicating a clinically relevant change in health status [18]. This minimal important difference for the EQ-5D summary index was determined using studies in different patient populations, including those with back pain [18]. The change in the physical component score of 8.2–9.6 derived using the SF-36v2 questionnaire during the open-label titration period was also above the 1.26–5.95 range for clinical relevance reported previously in patients recovering from lumbar spine surgery [19].

The bodily pain subscale of the SF-36v2 significantly favored patients randomized to continue ALO-02 compared with patients randomized to placebo during the double-blind treatment period, but other measures of HRQL, work productivity, and activity impairment did not show significant differences. This may be due to effects of ALO-02 persisting from the open-label period in patients randomized to placebo for the double-blind period. The specific design of randomized withdrawal trials, where the outcome of the double-blind treatment period is assessed on the worsening of pain with placebo relative to the active arm, may also contribute to the lack of significant differences between placebo and ALO-02 treatments [17].

When considering the overall study period, the bodily pain subscale of the SF-36v2 showed a significant difference. This is supported by the significant difference between ALO-02 and placebo in the WPAI:SHP measure of activity impairment due to low back pain during the overall study period.

There are limitations of this study that should be taken into consideration. First, the results may not be generalizable to a wider population because the placebo-controlled double-blind treatment period included a population enriched with treatment responders, whereas the open-label period of the study, which included all eligible patients, was uncontrolled. Second, improvements on various patient-reported outcome measures in the open-label titration period were based on comparison to baseline only, as this portion of the study by design was not placebo-controlled. Finally, the study was powered for the primary efficacy endpoint of NRS-Pain and not these secondary outcome measures.

## Conclusion

ALO-02 may improve HRQL, work productivity, and activity impairment in patients with chronic low back pain. Improvement was generally maintained during the randomized double-blind treatment period, although few treatment differences were observed. ALO-02 appears effective for pain relief and potentially also functional improvement in this chronic pain population. Further studies are needed to evaluate patient-reported functional outcomes as a primary or secondary endpoint in chronic pain populations.

## Abbreviations

ADO: Abuse-deterrent opioids
BPI-sf: Brief Pain Inventory-Short Form
CLBP: Chronic low back pain
EQ-5D-3L: EuroQol-5 Dimensions Health Questionnaire 3-Level version
HRQL: Health-related quality of life
IMMPACT: Initiative on Methods, Measurement, and Pain Assessment in Clinical Trials
ITT: Intent-to-treat
NRS: Numeric Rating Scale
PGA: Patient’s Global Assessment
RMDQ: Roland Morris Disability Questionnaire
SF-36v2: Short Form-36v2 Health Survey
VAS: Visual analog scale
WPAI:SHP: Work Productivity and Activity Impairment Questionnaire: Specific Health Problem

## References

[1] Institute of Medicine of the National Academies (2011). Relieving pain in America: a blueprint for transforming prevention, care, education, and research.

[2] Nahin RL (2015). Estimates of pain prevalence and severity in adults: United States, 2012. J Pain.

[3] Zorba PR (2010). Chronic pain management issues in the primary care setting and the utility of long-acting opioids. Expert Opin Pharmacother.

[4] de Leon-Casasola OA (2013). Opioids for chronic pain: new evidence, new strategies, safe prescribing. Am J Med.

[5] Chou R, Fanciullo GJ, Fine PG, Adler JA, Ballantyne JC, Davies P (2009). Clinical guidelines for the use of chronic opioid therapy in chronic noncancer pain. J Pain.

[6] Dowell D, Haegerich TM, Chou R (2016). CDC guideline for prescribing opioids for chronic pain--United States, 2016. JAMA.

[7] Rudd RA, Seth P, David F, Scholl L (2016). Increases in drug and opioid-involved overdose deaths - United States, 2010-2015. MMWR Morb Mortal Wkly Rep.

[8] Passik SD (2014). Tamper-resistant opioid formulations in the treatment of acute pain. Adv Ther.

[9] Rauck RL, Hale ME, Bass A, Bramson C, Pixton G, Wilson JG (2015). A randomized double-blind, placebo-controlled efficacy and safety study of ALO-02 (extended-release oxycodone surrounding sequestered naltrexone) for moderate-to-severe chronic low back pain treatment. Pain.

[10] Ware JE, Snow KK, Kosinski M, Gandek B (1993). SF-36 health survey manual and interpretation guide.

[11] Brooks R (1996). EuroQol: the current state of play. Health Policy..

[12] EuroQol Group (1990). EuroQol--a new facility for the measurement of health-related quality of life. Health Policy.

[13] Reilly MC, Zbrozek AS, Dukes EM (1993). The validity and reproducibility of a work productivity and activity impairment instrument. PharmacoEconomics.

[14] Acquadro C, Berzon R, Dubois D, Leidy NK, Marquis P, Revicki D (2003). Incorporating the patient's perspective into drug development and communication: an ad hoc task force report of the patient-reported outcomes (PRO) harmonization group meeting at the Food and Drug Administration, February 16, 2001. Value Health.

[15] U. S. Food and Drug Administration: Guidance for industry: analgesic indications: developing drug and biological products. 2013. https://www.fda.gov/downloads/drugs/guidancecomplianceregulatoryinformation/guidances/ucm384691.pdf. Accessed 17 June 2017.

[16] Dworkin RH, Turk DC, Peirce-Sandner S, Burke LB, Farrar JT, Gilron I (2012). Considerations for improving assay sensitivity in chronic pain clinical trials: IMMPACT recommendations. Pain.

[17] Katz N (2009). Enriched enrollment randomized withdrawal trial designs of analgesics: focus on methodology. Clin J Pain.

[18] Walters SJ, Brazier JE (2005). Comparison of the minimally important difference for two health state utility measures: EQ-5D and SF-6D. Qual Life Res.

[19] Copay AG, Glassman SD, Subach BR, Berven S, Schuler TC, Carreon LY (2008). Minimum clinically important difference in lumbar spine surgery patients: a choice of methods using the Oswestry disability index, medical outcomes study questionnaire short form 36, and pain scales. Spine J.

